# Expression of podoplanin in stromal fibroblasts plays a pivotal role in the prognosis of patients with pancreatic cancer

**DOI:** 10.1007/s00595-017-1559-x

**Published:** 2017-07-12

**Authors:** Kazuyoshi Hirayama, Hiroshi Kono, Yuuki Nakata, Yoshihiro Akazawa, Hiroyuki Wakana, Hisataka Fukushima, Hideki Fujii

**Affiliations:** First Department of Surgery, Faculty of Medicine, University of Yamanashi, 1110 Shimokato, Chuo, Yamanashi 409-3898 Japan

**Keywords:** Pancreatic cancer, Podoplanin

## Abstract

**Purpose:**

To investigate the role of podoplanin (PDPN) expression in invasive ductal carcinoma of the pancreas (IDCP) in humans.

**Methods:**

Tumor samples were obtained from 95 patients with IDCP. Immunohistochemical staining was done to evaluate the expression of PDPN in cancer tissues.

**Results:**

PDPN was detected predominantly in stromal fibroblasts, stained with α-smooth muscle actin. The cutoff value of PDPN-positive areas was calculated according to a histogram. There was no significant difference in clinicopathologic factors between patients with high vs. those with low PDPN expression. The high PDPN group showed significantly poorer disease-free and disease-specific survival rates than the low PDPN group. Among patients from the high PDPN group, those with lymph node metastases and those with a tumor larger than 20 cm in diameter had significantly poorer prognoses than similar patients from the low PDPN group. Multivariate Cox proportional hazards analysis indicated that a high expression of PDPN was an independent risk factor for disease-specific survival.

**Conclusions:**

PDPN expression in cancer-related fibrotic tissues is associated with a poor prognosis, especially in patients with large tumors or lymph node metastases.

## Introduction

Pancreatic cancer is associated with one of the poorest prognoses of any cancer because early detection is difficult and it progresses rapidly [1–3]. The number of deaths from pancreatic cancer is increasing in Japan. In fact, more than 30,000 deaths from pancreatic cancer in 2013 slightly exceeded the number of deaths from liver cancer in the same year [4].

Pancreatic fibrosis is one of the histopathologic findings at the time of the desmoplastic reaction associated with chronic pancreatitis or pancreatic cancer. Pancreatic stellate cells (PSCs) were first isolated and identified in the pancreas in 1998 [5, 6]. It was found that PSCs are similar to liver stellate cells and play a pivotal role in pancreatic fibrosis. In the normal pancreas, PSCs are quiescent and store cytoplasmic vitamin A-containing lipid droplets [7]. Inflammatory stimulation or signals from cancer cells activate PSCs, which develop a myofibroblast-like morphology and produce an extracellular matrix [7]. Activated PSCs secrete various growth factors and cytokines, such as fibroblast growth factor, transforming growth factor-β, stromal cell-derived factor 1, and interleukin-6 [8].

Podoplanin (PDPN), a 38-kDa type I transmembrane glycoprotein, is known as a marker of lymphatic endothelial cells [9]. In normal tissues, PDPN is expressed in kidney podocytes [10], alveolar type I cells [11], osteocytes [12], basal keratinocytes [13], and mesothelial cells [13]. Recently, PDPN was found in several other cancers, such as brain tumors [14], squamous cell carcinomas [15], germ cell tumors [16], and mesotheliomas [17]. It has been reported that PDPN expression is associated with malignancy in malignant astrocytic tumors [14]. PDPN is also found in some stromal fibroblasts, and an abundance of PDPN-positive stromal fibroblasts is associated with poor prognosis in lung adenocarcinoma, invasive breast cancer, and esophageal squamous cell carcinoma patients [18–20]. Since pancreatic cancer is rich in fibrous tissues, we investigated the correlation between PDPN expression in stromal fibroblasts in invasive ductal carcinoma of the pancreas (IDCP) and prognosis in humans.

## Materials and methods

### Patients and pancreatic cancer samples

Pancreatic cancer samples were obtained from 95 patients with IDCP, who underwent surgery at the University of Yamanashi Hospital between 1995 and 2013. Table 1 summarizes the clinicopathological characteristics of the patients. The histological diagnosis of the specimens was confirmed based on the criteria of the uploaded World Health Organization classification [21]. The stage was graded according to the Union for International Cancer Control (UICC) classification, 7th edition [22]. There were 57 men and 38 women, ranging in age from 46 to 83 years (median 70.0). One patient had stage 0 disease, five had stage IA, three had stage IB, 29 had stage IIA, 56 had stage IIB, and one had stage III. This study was approved by the Ethics Committee of Yamanashi University (approved no. 1565) and was performed in accordance with the ethical standards of the Declaration of Helsinki and its later amendments. Serum carcinoembryonic antigen, carbohydrate antigen 19-9, Duke pancreatic monoclonal antigen type 2, and s-pancreas antigen-1 levels were measured at least every 3 months. Computed tomography from the chest to pelvis was performed at least every 6 months. Survival was measured from the time of pancreatic resection until death or censor. The follow-up duration ranged from 3 to 191 months.

**Table 1:** Clinicopathological characteristics of the 95 patients with invasive ductal carcinoma of the pancreas

Variables	Number of patients	%
PDPN positive area (%)	11.83 (0.45–36.29)	
Age (years)	70.0 (46–83)	
Sex		
Male	57	60
Female	38	40
Histologic grade		
G1	28	29.5
G2	55	57.9
G3	12	12.6
Tumor size (mm)	27.9 (3–90)	
Microscopic venous invasion		
Yes	83	87.4
No	12	12.6
Microscopic lymphatic vessel invasion		
Yes	70	73.7
No	25	26.3
UICC T category		
0	1	1.1
1	6	6.3
2	3	3.2
3	84	88.4
4	1	1.1
UICC N category		
0	39	41.1
1	56	58.9
UICC stage		
0	1	1.1
IA	5	5.3
IB	3	3.2
IIA	29	30.5
IIB	56	58.9
III	1	1.1

*UICC* Union for International Cancer Control, *PDPN* podoplanin

### Immunohistochemistry for D2-40 and α-SMA

**Fig. 1:**
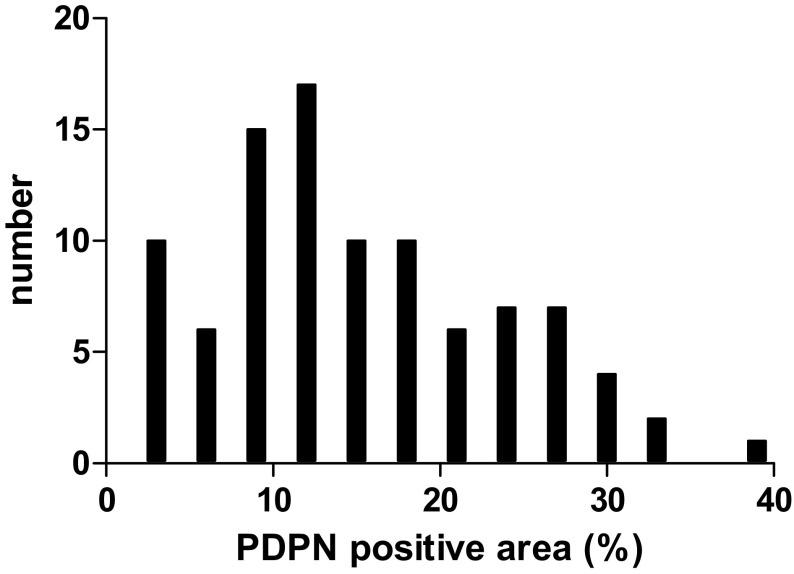
Histogram of podoplanin (PDPN) expression. A histogram was created to calculate the cutoff value of the PDPN-positive area

Formalin-fixed, paraffin-embedded tissue specimens were cut into 4-µm sections. Each section was mounted on a silane-coated glass slide, deparaffinized, and treated in antigen retrieval solution for 15 min at 120 °C using Dako REAL Target Retrieval Solution (Dako, Carpentaria, CA, USA). Endogenous peroxidase was quenched by incubation at room temperature in 0.3% H_2_O_2_, followed by rinsing with phosphate-buffered saline. Endogenous biotin was quenched using the Dako Biotin Blocking System (Dako). Sections were blocked using 5% normal blocking serum for 20 min. Mouse monoclonal to D2-40 antibodies (1:40; Abcam, Cambridge, UK) were applied overnight at 4 °C to stain PDPN. Rabbit polyclonal to α-smooth muscle actin antibodies (α-SMA, 1:200; Abcam) were applied for 2 h at room temperature. Following incubation, immunoperoxidase staining was completed using a Vectastain ABC elite kit (Vector Laboratories, Burlingame, CA, USA) and 3,3′-diaminobenzidine-tetrachloride as a chromogen. The D2-40-positive area was calculated from three different (100×) fields and is expressed as a percentage of the total area of the field using PhotoShop and Image J software. To calculate the cutoff value of the PDPN-positive area, a histogram was created (Fig. 1).

### Statistical analysis

Data are expressed as mean ± standard error of the mean (SEM). Comparisons between two groups were assessed using the unpaired *t* test. Associations between different categorical variables were assessed using the *χ* ^2^ test. Survival rates were calculated using the Kaplan–Meier method, and significant differences in survival were determined by the log-rank test. The Cox proportional hazards model served for uni- and multivariable survival analysis. *p* < 0.05 was considered significant.

## Results

### Analysis of PDPN expression in pancreatic cancer by immunohistochemistry for D2-40

**Fig. 2:**
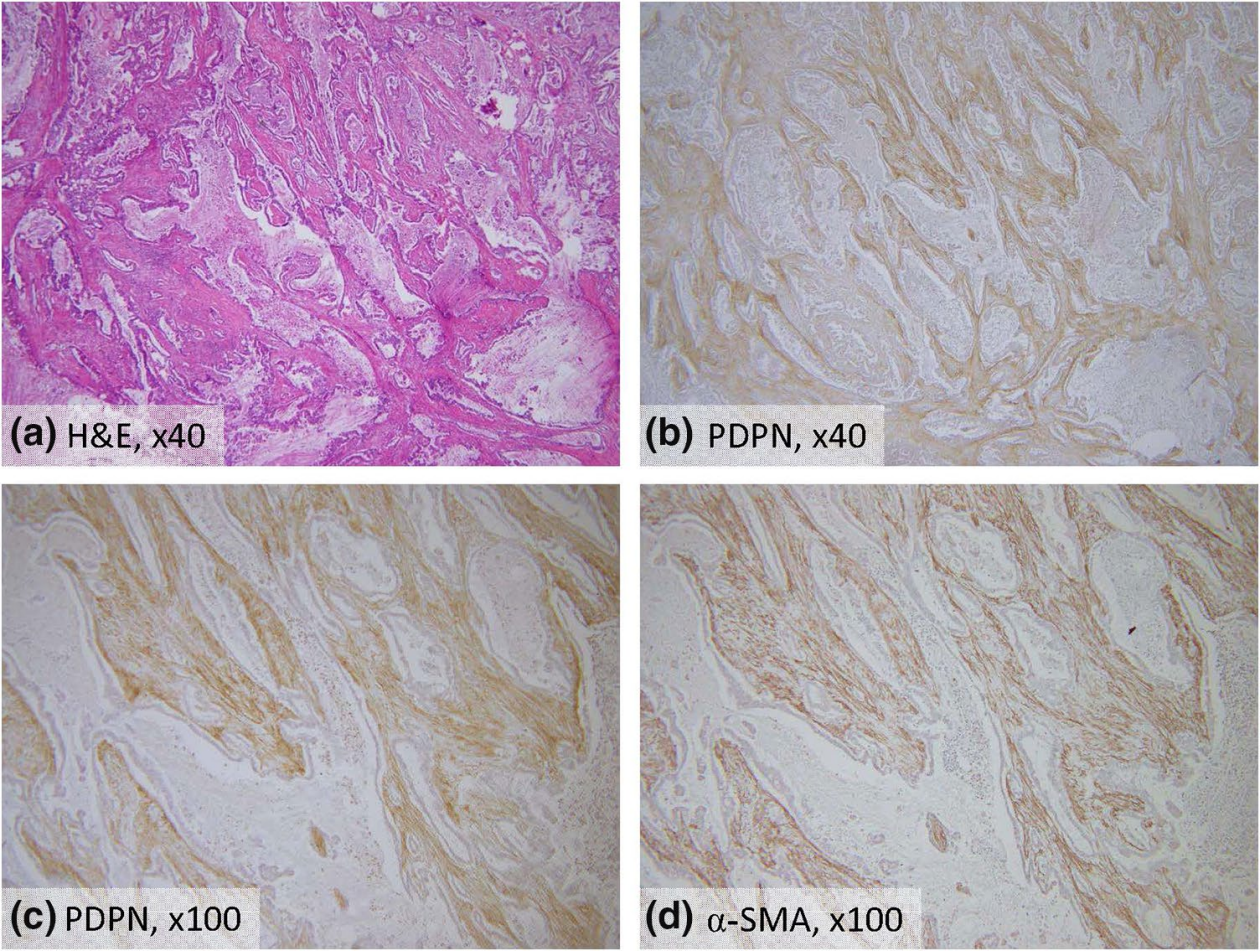
Hematoxylin-eosin (H&E) and immunohistochemical staining for podoplanin (PDPN) and αSMA in invasive ductal carcinoma of the pancreas. Immunohistochemical staining for PDPN (**b**, **c**) and αSMA (**d**) was performed as described in “Materials and methods”. Representative photomicrographs are shown. PDPN-expressing areas in the pancreatic cancer also expressed α-SMA. Original magnification, ×40 (**a**, **b**) and ×100 (**c**, **d**)

We performed immunohistochemical staining for D2-40 and α-SMA to evaluate PDPN expression in pancreatic cancer (Fig. 2). The areas that expressed PDPN in the pancreatic cancer also expressed α-SMA, a marker of stromal fibroblasts [23]. The PDPN-positive area in the pancreatic cancers ranged from 0.45 to 36.29% (median 11.83). A histogram was produced to establish the cutoff value of the PDPN-positive area, which was 11.83%, being the median value of the PDPN-positive areas. Patients with high expression of PDPN accounted for 52.6% of the patients (*n* = 50). There was no significant difference in clinicopathologic factors, except for the PDPN-positive area, between the group with high PDPN expression (high PDPN group) and the group with low PDPB expression (low PDPN group; Table 2).

**Table 2:** Relationships between podoplanin expression and clinicopathologic factors

Variables	High PDPN expression		Low PDPN expression		*p* value
	*n* = 50 (52.6%)		*n* = 45 (47.4%)		
	Number of patients	%	Number of patients	%	
PDPN positive area (%)	20.21		6.74		<0.0001
Age (years)	69.2		69		0.926
Sex					0.675
Male	29	58	28	62.2	
Female	21	42	17	37.8	
Histologic grade					0.241
G1	12	24	16	35.6	
G2	33	66	22	48.9	
G3	5	10	7	15.6	
Tumor size (mm)	28.3		27.5		0.791
Microscopic venous invasion					0.152
Yes	46	92	37	82.2	
No	4	8	8	17.8	
Microscopic lymphatic vessel invasion					0.141
Yes	40	80	30	66.7	
No	10	20	15	33.3	
UICC T category					0.569
0	0	0	1	2.2	
1	4	8	2	4.4	
2	1	2	2	4.4	
3	44	88	40	88.9	
4	1	2	0	0	
UICC N category					0.826
0	20	40	19	42.2	
1	30	60	26	57.8	
UICC stage					0.521
0	0	0	1	2.2	
IA	4	8	1	2.2	
IB	1	2	2	4.4	
IIA	14	28	15	33.3	
IIB	30	60	26	57.8	
III	1	2	0	0	
Recurrence					0.409
Yes	33	66	26	57.8	
No	17	34	19	42.2	

Statistical significances were calculated using the student’s *t* test or the *χ* ^2^ test

*PDPN* podoplanin

### Correlation between PDPN expression in pancreatic cancer and prognosis

**Fig. 3:**
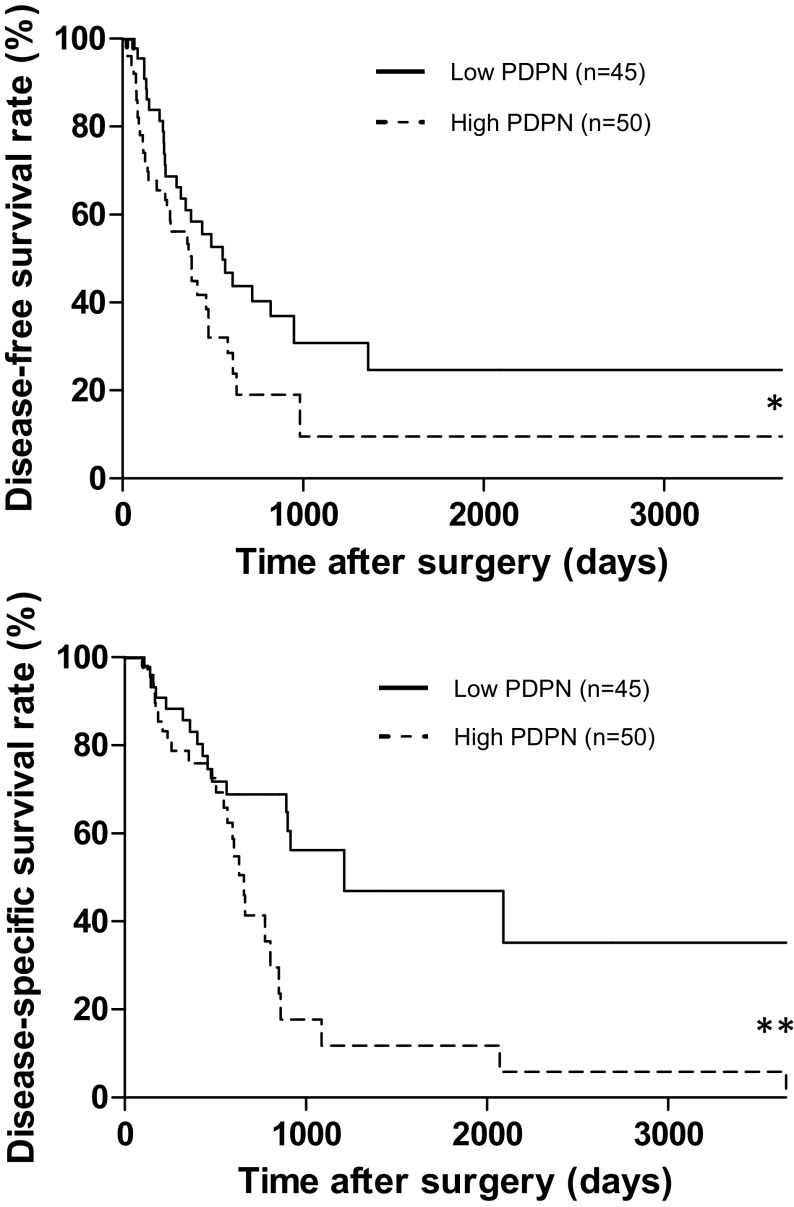
Comparison of survival curves by the Kaplan–Meier survival method for invasive ductal carcinoma of the pancreas according to podoplanin expression. Patients with high PDPN expression had a significantly poorer prognosis than those with low PDPN expression. *p < 0.05 and **p < 0.01 vs. low PDPN expression by the log-rank test

**Fig. 4:**
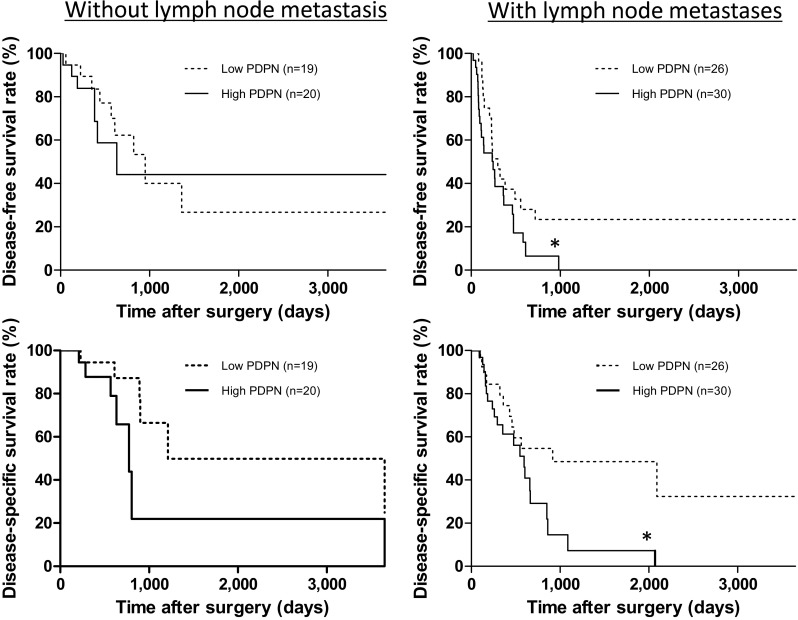
Comparison of survival curves by the Kaplan–Meier survival method for invasive ductal carcinoma of the pancreas according to lymph node metastasis. In patients without lymph node metastasis, there was no significant difference in the survival rate according to PDPN expression. In patients with lymph node metastases, patients with high PDPN expression had a significantly poorer prognosis than those with low PDPN expression. *p < 0.05 vs. low PDPN expression without lymph node metastasis by the log-rank test

**Fig. 5:**
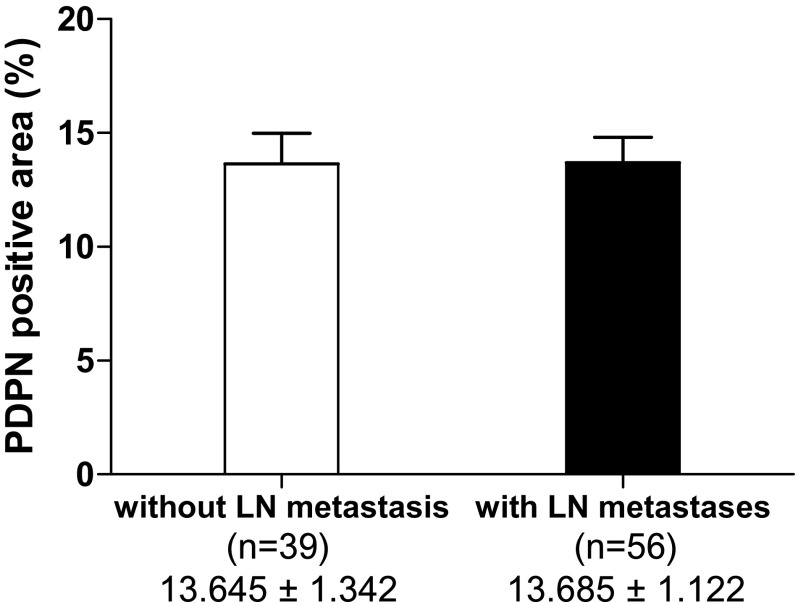
Analysis of the ratio of the podoplanin (PDPN)-positive area between patients with vs. those without lymph node metastasis. The ratio of the PDPN-positive area between patients with and those without LN metastasis is shown. Data represent the mean ± standard error of the mean. There was no significant difference according to PDPN expression by the unpaired *t* test. *LN* lymph node

The high PDPN group had significantly poorer disease-free survival (DFS) and disease-specific survival (DSS) rates than the low PDPN group (Fig. 3). The median survival times for the high and low PDPN groups were 659 and 1212 days, respectively. We then analyzed survival according to the presence of lymph node metastasis. In the patients without lymph node metastasis, there was no significant difference in DFS or DSS according to PDPN expression, but in those with lymph node metastases, the high PDPN group had significantly poorer DFS and DSS rates than the low PDPN group (Fig. 4). There was no significant difference in the PDPN-positive area between patients with and those without lymph node metastasis (Fig. 5).

**Fig. 6:**
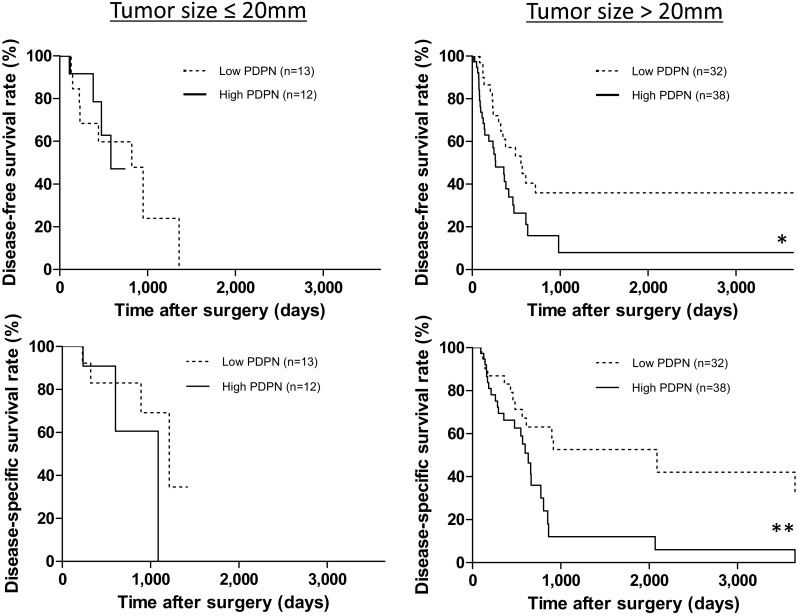
Comparison of survival curves by Kaplan–Meier survival method for invasive ductal carcinoma of the pancreas according to tumor size. In patients with tumors ≤20 mm, there was no significant difference in the survival rate according to PDPN expression. In patients with tumors >20 mm, those with high PDPN expression had a significantly poorer prognosis than those with low PDPN expression. *p < 0.05 and **p < 0.01 vs. low PDPN expression in patients with tumors >20 mm by the log-rank test

**Fig. 7:**
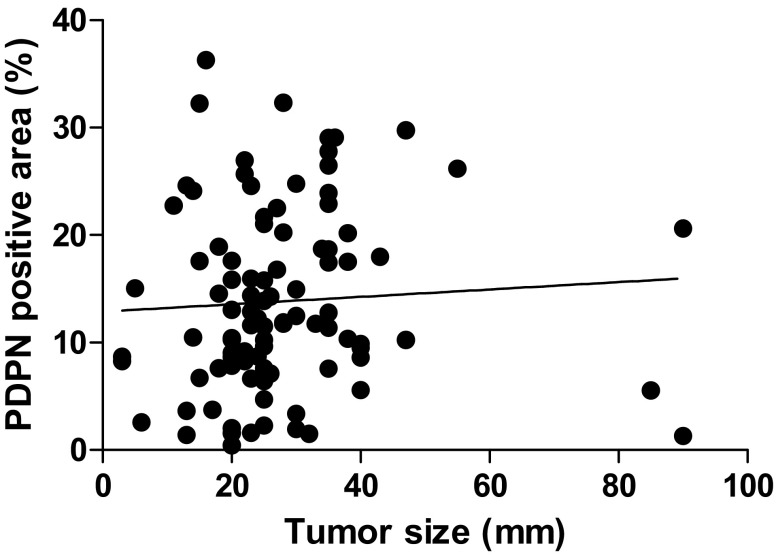
Immunohistochemical staining for podoplanin (PDPN) in invasive ductal carcinoma of the pancreas (IDCP). Immunohistochemical staining for PDPN was performed as described in “Materials and methods”. A scatter plot of the ratio of the PDPN-positive area in IDCP and tumor size is shown. There was no correlation

Focusing on the tumor size, in patients with tumors ≤20 mm, there was no significant difference in DFS or DSS according to PDPN expression, but in those with tumors >20 mm, the high PDPN group had significantly poorer DFS and DSS rates than the low PDPN group (Fig. 6). There was no correlation between PDPN expression and tumor size (Fig. 7).

### Prognostic factors

We adopted factors found to be significant by univariate analysis, based on the multivariate Cox proportional hazards analysis. A high expression of PDPN was an independent risk factor for DSS (relative risk (RR) = 2.153, *p* = 0.022) and tumor size >20 mm was an independent risk factor for both DFS (RR = 2.514, *p* = 0.013) and DSS (RR = 2.535, *p* = 0.032; Table 3).

**Table 3:** Univariate and multivariate survival analysis (Cox regression model) of clinicopathologic factors and podoplanin expression

Factors	DFS				DSS			
	Univariate	Multivariate			Univariate	Multivariate		
	*p* value	Hazard ratio	95% CI	*p* value	*p* value	Hazard ratio	95% CI	*p* value
PDPN positive area: <11.83 vs. ≥11.83%	0.047			NS	0.006	2.153	1.116–4.153	0.022
Age: <65 vs. ≥65 years	0.547			NA	0.842			NA
Sex: female vs. male	0.663			NA	0.528			NA
Histologic grade: G1 vs. G2 and G3	0.189			NA	0.003	2.592	1.159–5.795	0.020
Tumor size: ≤20 vs. >20 mm	<0.001	2.514	1.211–5.220	0.013	0.002	2.535	1.081–5.943	0.032
Microscopic venous invasion: no vs. yes	0.366			NA	0.980			NA
Microscopic lymphatic vessel invasion: no vs. yes	0.126			NA	0.651			NA
UICC T category: T0, T1, and T2 vs. T3 and T4	0.014			NS	0.047			NS
UICC N category: N0 vs. N1	<0.001			NS	0.021			NS
UICC stage: 0-IIA vs. IIB/III	<0.001	2.223	1.203–4.105	0.011	0.011			NS

*DFS* disease-free survival, *DSS* disease-specific survival, *CI* confidence interval, *NA* not adopted, *NS* not significant, *UICC* Union for International Cancer Control, *PDPN* podoplanin

## Discussion

The expression of stromal fibroblasts markers, including α-SMA, vimentin, desmin, fibroblast specific protein-1, and fibroblast-activation protein, is reported to vary depending on the local microenvironment of tumors [23]. Apte et al. reported that α-SMA-positive activated PSC also expressed glial fibrillary acidic protein or/and desmin in pancreatic cancer [24]. Recent studies investigating the roles of PSCs in pancreatic cancer have identified a mechanism of interaction among proliferation, invasion, and metastasis [7, 8, 25–31]. In the present study, PDPN-expressing stromal cells in pancreatic cancer also expressed α-SMA, suggesting that they were most likely derived from PSCs (Fig. 2).

PDPN expression in stromal fibroblasts in pancreatic cancer was reported to be associated with lymphatic invasion, vascular invasion, the tumor size, histological grade, UICC classification T stage, and a shorter survival period [32]. Those results indicate that PDPN expression is associated with the progression of carcinoma in local recurrence, hematogenous metastasis, and lymphogenous metastasis [32, 33]. The present study found no significant difference in clinicopathologic factors between the high and low PDPN groups (Table 2). There were also no significant differences between PDPN expression and the incidence of lymph node metastases or the size of tumors (Figs. 5, 7). PDPN could be related to tumor growth, leading to poor prognosis, and its expression might be determined by the genotype of each tumor. Importantly, DFS and DSS were significantly poorer in the high PDPN group (Fig. 3). Thus, as PDPN expression is involved in the prognosis of pancreatic cancer patients, it may be a useful marker to identify patients with a poor prognosis after surgery.

Based on previous reports, lymphatic invasion, vascular invasion, tumor size, pathological grade, and UICC classification T stage are all useful for pathologic staging; however, no effective molecular targeting therapy after surgery has been established. In this regard, PDPN may be a useful and effective molecular target for therapy. Indeed, Kato et al. developed a cancer-specific monoclonal antibody against human PDPN, which reacted with PDPN-expressing cancer cells, but not with normal cells [34]. Although this antibody is promising for molecular targeting therapy against PDPN-expressing cancers, PDPN is expressed only on the stromal fibroblasts surrounding tumors in pancreatic cancer. Conversely, Suzuki-Inoue et al. reported that PDPN expressed in cancer cells promotes platelet aggregation and it may also be involved in migration, invasion, metastasis, and the malignant progression of cancer cells [35]. Thus, PDPN expressed in stromal fibroblasts may be involved in cancer progression, leading to a poor prognosis via mechanisms of multiple growth factors derived from activated platelets. Further investigations are needed to realize useful targeting therapy against PDPN in pancreatic cancer.

## Conclusions

Podoplanin expression in stromal fibroblasts is associated with the poor prognosis of patients with large tumors or lymph node metastases of pancreatic cancer. Our findings suggest that patients with high expression of PDPN should be followed-up more closely after surgery. PDPN may become an important target of therapy for pancreatic cancer.

## References

[1] Yamada D, Eguchi H, Asaoka T, Tomohara H, Noda T, Wada H (2017). The basal nutritional state of PDAC patients is the dominant factor for completing adjuvant chemotherapy. Surg Today.

[2] Fukuda Y, Yamada D, Eguchi H, Iwagami Y, Noda T, Asaoka T (2017). A novel preoperative predictor of pancreatic fistula using computed tomography after distal pancreatectomy with staple closure. Surg Today.

[3] Tomihara H, Eguchi H, Yamada D, Gotoh K, Kawamoto K, Wada H (2017). Preoperative chemoradiotherapy does not compromise the feasibility of adjuvant chemotherapy for patients with pancreatic ductal adenocarcinoma. Surg Today.

[4] Ministry of Health, Labour and Welfare. Vital statistics Japan. Center for Cancer Control and Information Services, National Cancer Center, Japan. 2015.

[5] Apte MV, Haber PS, Applegate TL, Norton ID, McCaughan GW, Korsten MA (1998). Periacinar stellate shaped cells in rat pancreas: identification, isolation, and culture. Gut.

[6] Bachem MG, Schneider E, Gross H, Weidenbach H, Schmid RM, Menke A (1998). Identification, culture, and characterization of pancreatic stellate cells in rats and humans. Gastroenterology.

[7] Omary MB, Lugea A, Lowe AW, Pandol SJ (2007). The pancreatic stellate cell: a star on the rise in pancreatic diseases. J Clin Investig.

[8] Vonlaufen A, Phillips PA, Xu Z, Goldstein D, Pirola RC, Wilson JS (2008). Pancreatic stellate cells and pancreatic cancer cells: an unholy alliance. Cancer Res.

[9] Breiteneder-Geleff S, Soleiman A, Kowalski H, Horvat R, Amann G, Kriehuber E (1999). Angiosarcomas express mixed endothelial phenotypes of blood and lymphatic capillaries: podoplanin as a specific marker for lymphatic endothelium. Am J Pathol.

[10] Matsui K, Breitender-Geleff S, Soleiman A, Kowalski H, Kerjaschki D (1999). Podoplanin, a novel 43-kDa membrane protein, controls the shape of podocytes. Nephrol Dial Transplant.

[11] Vanderbilt JN, Dobbs LG (1998). Characterization of the gene and promoter for RTI40, a differentiation marker of type I alveolar epithelial cells. Am J Respir Cell Mol Biol.

[12] Wetterwald A, Hofstetter W, Cecchini MG, Lanske B, Wagner C, Fleish H (1996). Characterization and cloning of the E11 antigen, a marker expressed by rat osteoblasts and osteocytes. Bone.

[13] Schacht V, Dadras SS, Johnson LA, Jackson DG, Hong YK, Detmar M (2005). Up-regulation of the lymphatic marker podoplanin, a mucin-type transmembrane glycoprotein, in human squamous cell carcinomas and germ cell tumors. Am J Pathol.

[14] Mishima K, Kato Y, Kaneko MK, Nishikawa R, Hirose T, Matsutani M (2006). Increased expression of podoplanin in malignant astrocytic tumors as a novel molecular marker of malignant progression. Acta Neuropathol.

[15] Martín-Villar E, Scholl FG, Gamallo C, Yurrita MM, Muñoz-Guerra M, Cruces J (2005). Characterization of human PA2.26 antigen (T1α-2, podoplanin), a small membrane mucin induced in oral squamous cell carcinoma. Int J Cancer.

[16] Kato Y, Sasagawa I, Kaneko M, Osawa M, Fujita N, Tsuruo T (2004). Aggrus: a diagnostic marker that distinguishes seminoma from embryonal carcinoma in testicular germ cell tumors. Oncogene.

[17] Kimura N, Kimura I (2005). Podoplanin as a marker for mesothelioma. Pathol Int.

[18] Kawase A, Ishii G, Nagai K, Ito T, Nagano T, Murata Y (2008). Podoplanin expression by cancer associated fibroblasts predicts poor prognosis of lung adenocarcinoma. Int J Cancer.

[19] Schoppmann SF, Berghoff A, Dinhof C, Jakesz R, Gnant M, Dubsky P (2012). Podoplanin-expressing cancer-associated fibroblasts are associated with poor prognosis in invasive breast cancer. Breast Cancer Res Treat.

[20] Tong L, Yuan S, Feng F, Zhang H (2012). Role of podoplanin expression in esophageal squamous cell carcinoma: a retrospective study. Dis Esophagus.

[21] Hruban RH, Klöppel G, Boffetta P, Maitra A, Hiraoka N, Offerhaus GJA, et al. Ductal adenocarcinoma of the pancreas. In: WHO classification of tumors of the digestive system. World Health Organization; 2010. p. 281–91.

[22] Edge SB, Byrd DR, Compton CC, Fritz AG, Greene FL, Trotti A (2010). Exocrine and endocrine pancreas, AJCC cancer staging manual.

[23] Kallui R, Zeisberg M (2006). Fibroblasts in cancer. Nat Rev Cancer.

[24] Apte MV, Park S, Phillips PA, Santucci N, Goldstein D, Kumar RK (2004). Desmoplastic reaction in pancreatic cancer: role of pancreatic stellate cells. Pancreas.

[25] Algul H, Treiber M, Lesina M, Schmid RM (2007). Mechanisms of disease: chronic inflammation and cancer in pancreas—a potential role for pancreatic stellate cells?. Nat Clin Pract Gastroenterol Hepatol.

[26] Aoki H, Ohnishi H, Hama K, Ishijima T, Satoh Y, Hanatsuka K (2006). Autocrine loop between TGF-beta1 and IL-1 beta through Smad3- and ERK-dependent pathways in rat pancreatic stellate cells. Am J Physiol Cell Physiol.

[27] Bachem MG, Zhou S, Buck K, Schneiderhan W, Siech M (2008). Pancreatic stellate cells—role in pancreas cancer. Langenbecks Arch Surg.

[28] Hwang RF, Moore T, Arumugam T, Ramachandran V, Amos KD, Rivera A (2008). Cancer-associated stromal fibroblasts promote pancreatic tumor progression. Cancer Res.

[29] Miyamoto H, Murakami T, Tsuchida K, Sugino H, Miyake H, Tashiro S (2004). Tumor-stroma interaction of human pancreatic cancer: acquired resistance to anticancer drugs and proliferation is dependent on extracellular matrix proteins. Pancreas.

[30] Sato N, Maehara N, Goggins M (2004). Gene expression profiling of tumor-stromal interaction between pancreatic cancer cells and stromal fibroblasts. Cancer Res.

[31] Vonlaufen A, Joshi S, Qu C, Phillips PA, Xu Z, Parker NR (2008). Pancreatic stellate cells: partners in crime with pancreatic cancer cells. Cancer Res.

[32] Shindo K, Aishima S, Ohuchida K, Fujiwara K, Fujino M, Mizuuchi Y (2013). Podoplanin expression in cancer-associated fibroblasts enhances tumor progression of invasive ductal carcinoma of the pancreas. Mol Cancer.

[33] Sato D, Tsuchikawa T, Mitsuhashi T, Hatanaka Y, Marukawa K, Morooka A (2016). Stromal paladin expression is an independent prognostic factor in pancreatic ductal adenocarcinoma. PLoS One.

[34] Kato Y, Kaneko MK (2014). A cancer-specific monoclonal antibody recognizes the aberrantly glycosylated podoplanin. Sci Rep.

[35] Suzuki-Inoue K, Kato Y, Inoue O, Kaneko MK, Mishima K, Yatomi Y (2007). Involvement of the snake toxin receptor CLEC-2, in podoplanin-mediated platelet activation, by cancer cells. J Biol Chem.

